# Community-Based Professional Development in Core Facilities: ABRF and CTLS Case Studies

**DOI:** 10.7171/001c.166059

**Published:** 2026-09-01

**Authors:** Joshua Z. Rappoport, Heather M. Olson, Ilaria Guerini, Luellen Fletcher, Ian A. Brewis, Laura J. Lewis-Tuffin

**Affiliations:** 1 Office of the Vice Provost for Research Boston College; 2 Biological Sciences Division Pacific Northwest National Laboratory; 3 Scientific Operations Human Technopole; 4 Path BioResource (retired from) Perelman School of Medicine, University of Pennsylvania; 5 School of Medicine Cardiff University; 6 Office of Core Shared Services Mayo Clinic

**Keywords:** core facilities, job-shadowing, mentoring, professional development programs

## Abstract

The shift toward the shared scientific resource model (core facilities) has resulted in a growing scientific workforce whose roles extend beyond technical expertise to include customer service, business management, and leadership soft skills. Competency in these areas is critical to success, but formal training is rare, making mentoring, coaching, and job shadowing essential tools for professional development and career sustainability within core facilities. Two professional associations, the Association for Biomolecular Resource Facilities (ABRF) and Core Technologies for Life Sciences (CTLS), have developed mentoring and job shadowing programs to address these needs. The ABRF Mentoring Program, launched in 2016, has undergone multiple iterations, revealing key lessons around flexibility, mentor recruitment, expectation setting, relationship building, and the limits of highly automated platforms. In parallel, the CTLS Mentoring Program, initiated in 2022, emphasizes low barriers to participation, informality, and external perspectives. Both programs note reciprocal learning between mentors and mentees as a valuable outcome to program participation. Complementing mentorship, the CTLS Job Shadowing Programme provides short, immersive, peer-to-peer experiential learning focused on facility operations and management, yielding bidirectional benefits for both visitors and hosts. These initiatives are valuable member benefits for their respective professional associations. Their success highlights the important role of human connection in professional growth and demonstrates that lightly structured, community-driven programs with continuous feedback can effectively support diverse career paths in core facilities. As core facility careers continue to expand, these programs will remain critical to building a resilient, skilled, and interconnected research infrastructure workforce.

## Introduction

The concept of core facilities is relatively new in scientific fields. While the first shared facilities were initiated in the late 1970s and early 1980s, the idea didn’t gain real traction until the 1990s when a high throughput revolution made automated technologies and techniques commonplace. Increased output came with hefty price tags to purchase and maintain such equipment. This led many scientists to share these resources or reach out to other institutions to utilize the resources that they could not afford.^1^ Gradually, such equipment became consolidated in core facilities, which became centers for scientific innovation that often combined multiple technologies into streamlined workflows.^2^ Today, the staff of core facilities must be scientifically and technologically skilled, demonstrate excellent customer service skills, and be familiar with business management practices.

While it is exciting to be on the cutting edge of research, formal training in customer service and business “soft skills” are typically nonexistent for scientists who suddenly find themselves running a core facility (often alone). In traditional academic settings, mentoring is often considered to be helpful but not critical for advancing one’s career. In the case of scientific core facilities, such relationships are often essential for success in core facility administrative and leadership roles. Mentoring, coaching, and job shadowing are critical and vital tools for scientists and administrators who must navigate such unfamiliar territory as core facility management, rate-setting for their services, or obtaining sufficient funding to fully staff their facilities. Being able to reach out and learn from peers through professional organizations such as the Association for Biomolecular Resource Facilities (ABRF) or Core Technologies for Life Sciences (CTLS) has saved many core facilities from potentially bad outcomes. Within these organizations, mentoring options, including peer-to-peer mentoring, group mentoring, job shadowing, and coaching, are key professional development benefits for members. In many parts of Europe, job shadowing, a compressed combination of mentoring, training, and coaching, is offered as a tool to learn firsthand all aspects of how to run a core facility.

In the world of professional development, mentoring and coaching are considered to be very different. Mentoring is typically a longer-term relationship (six months is most common) between someone who has more experience in a field (the mentor) and someone who is looking for guidance and knowledge about that same field (the mentee). Less formal than coaching, mentoring is a broader working relationship focused on professional and personal development. Mentors typically don’t have formal counseling training, but they come to the relationship willing to help generate an environment where the mentee can gain insights, build networks, or explore career paths. Often, mentors state that they also learn from their mentees, indicating the relationship can be a two-way street that fosters reciprocal growth. Shorter-term mentoring relationships such as group mentoring workshops are often focused on a specific topic. Speed mentoring is a technique like speed dating where a mentee can ask the same question to multiple mentors in a short period of time, obtaining multiple points of view for the same issue. Both group mentoring and speed mentoring can lead to longer-term mentoring relationships.

Coaching is focused on specific skill development and performance improvement, helping scientists achieve objectives within a defined timeframe. Coaching is often shorter term (one to two months), and coaches often have formal training. A certified coach employs structured methodologies, asking probing questions to facilitate personal growth and accountability. The coach is not expected to have technical subject expertise in common with the person being coached, which forces that person to generate their own ideas and knowledge. Coaching is often focused on career, performance, or leadership development, with a specific and measurable goal being defined at the beginning of the relationship. The coach definitively leads the progress, and it is a one-way relationship between the two participants.

Job shadowing is broadly based upon the process of “secondment,” whereby staff with a specific role benefit from experiential learning by working in a different office, division, or even institution. CTLS has created a Job Shadowing Program that specifically supports the professional development of core facility staff through short-term experience in a similar facility at a different institution, facilitating the exchange of knowledge and expertise among peers. A core facility hosts a qualified visitor from another institution, which encourages collaboration on specific tasks and activities relevant to facility operations or management. Each visitor is paired with an experienced staff member who offers guidance and support throughout the daily workflow. This allows facilities to initiate or reinforce collaborations, share best practices, and learn from one another, with the goal of improving efficiency in multiple areas of their operations and management. The visits are often two to five working days and can cover a broad range of topics (e.g., facility organization, operation and management, safety, quality control, facility technologies). The visitor learns how another organization is operated, bringing improvements and new techniques back to their facility. The host facility benefits by creating stronger collaborations and getting an influx of new ideas around research, operations, and management from the visitor.

As core facilities have become the new model for performing scientific research, the concept of scientific career paths within cores instead of careers as independent researchers has grown. Developing one’s career within a core can have many facets. Depending on the institution, in a well-established core, a person can start as a technician and work their way up into administration of a single core or even become the administrator of a group of cores. Additionally, one can pursue continuous technical improvement within a core and advance to becoming a key contributor to combinatorial research within multiple cores. The “flavor” of advancement is often defined by the facility, but there can be flexibility. Creative core staff may define their own career path or propose new organizational roles when they identify needs that they are well-suited to meet.

This perspective focuses on professional development initiatives realized through the tireless efforts of the members and leadership of the ABRF and CTLS, two nonprofit associations working to support core facility scientists and administrators. In particular, the ABRF Mentoring Program and the CTLS Mentoring and Job Shadowing Programs are designed to support the wide variety of roles and career stages found in the core facility world. Descriptions of these programs can be found in the following sections, including discussion of challenges and successes in serving a hugely varied set of professionals.

## ABRF Mentoring Program

The ABRF Career Development Committee (CDC) created its mentoring program in 2016. Since the program’s inception, the committee has embraced a continuous improvement approach, with the goal of providing efficient and sustainable value to program participants. As a result, the program has gone through several iterations.

### Program Origins

The ABRF Mentoring Program^3^ grew out of the observation that structured connections could help colleagues navigate career transitions and day-to-day challenges in core facility environments. In 2015, Deb McMillen shared with the CDC how intentional outreach to her professional network provided unusually candid, practical guidance during her own career transition – an experience that suggested the value of a community-wide framework for ABRF members. This was the seed idea that CDC members grew into the ABRF Mentoring Program.

From the beginning, the CDC set two anchors for the program’s design: (1) flexibility for mentees to define their own objectives, ranging from technical skills, leadership, operations, and interpersonal dynamics; and (2) explicit attention to diversity, inclusivity, and culture within mentoring relationships, recognizing the breadth of roles and disciplines across ABRF. The aim was to cultivate mentoring as an enduring aspect of membership rather than a time-limited program.

To confirm demand for the program, the CDC surveyed the community (170 respondents) and found broad support for a CDC-mediated effort, with a surprisingly strong emphasis on core management topics. To sharpen the model, the committee performed literature reviews and sought external perspectives, consulting with established programs (e.g., the National Council of University Research Administrators) and the University of New Mexico (UNM) Mentoring Institute (Director Nora Dominguez). With support from the ABRF Executive Board (EB), CDC members Anitha Chennat and Deb McMillen attended the 2016 UNM Mentoring Conference to learn about mentoring styles and platforms from other established programs. A key takeaway was the ethos of iterative improvement grounded in frequent participant feedback. As a result, the ABRF Mentoring Program includes participant surveys at multiple points during each mentor-mentee pairing, using that input to guide program changes.

Two complementary pilot programs in 2016 tested feasibility and logistics. A group mentoring cohort led by Phil Hockberger, which engaged Chicago-area core directors as both mentors and mentees, demonstrated the practical limits of site-bound, in-person cohorts for a geographically distributed community such as ABRF. In parallel, a five-pair, one-to-one pilot across several specialties validated the goal-driven design and highlighted the need to recruit and sustain a mentor pool large enough to support good matches across diverse mentee goals.

### Exploration of Program Infrastructure Options

With lessons from the pilot programs, the CDC next explored hosted infrastructures to support customization, matching, and routine feedback at scale. The program first partnered with the National Research Mentoring Network (NRMN), leveraging its structured, guided-virtual environment with the creation of an ABRF subgroup. Initial pairings were made manually by CDC members Luellen Fletcher and Anitha Chennat. As participation grew, the CDC collaborated with NRMN to fine-tune the inputs used for matching, streamline communications, and tailor prompts and surveys for a professional audience.

By 2019, multiple cohorts spanned ABRF subfields and career stages, underscoring sustained member interest. At the same time, participant feedback indicated opportunities to simplify how users navigated the host environment. In response, the CDC reassessed how best to balance usability, customization, and administrative oversight.

In 2021, the CDC switched to a portal hosted by sfgMentorNet to gain direct configuration control. This change enabled rolling admissions, keyword-based matching (initiated by mentees or administrators), automated survey deployment, analytics, and international participation. Early interest was strong and was supported by an expanded mentor pool and a steady stream of mentee enrollments.

Over time, familiar engagement challenges emerged that are common across many online mentoring contexts: some participants did not complete profiles; many mentees delayed initiating matches; and pairs often preferred to move conversations outside the portal. These patterns limited the usefulness of in-platform analytics and made it harder to gauge relationship quality. To address this, administrators introduced concise community standards for roles, participation, and communication, and they began confirming mentor availability before finalizing matches in the hopes that these process adjustments would clarify expectations while preserving responsiveness.

As virtual meeting norms matured during the COVID-19 era, ABRF reintroduced the idea of a scalable community-oriented space for shared problem solving by piloting a monthly group mentoring forum (facilitated by ABRF Executive Director Ken Schoppman). In addition, by expanding an event that originated in an ABRF Chapter meeting, the CDC has facilitated a speed mentoring session at every annual ABRF meeting since 2020. With these alternatives in place and committee membership transitions ahead, the CDC concluded the sfgMentorNet contract in late 2022 and paused one-to-one mentoring operations to refocus on a model that best matched member behavior and needs.

In 2025, the program relaunched with a streamlined, participant-centered format. It included: a combined intake mechanism for mentors and mentees; brief facilitator interviews to clarify mentee goals; facilitator attendance for the first five minutes of an initial meeting to ensure pairs have a strong start and shared expectations; and concise two- and six-month check-ins focused on progress against mentee-defined goals. Early results (several completed pairings and high survey response rates) suggest that a light-touch infrastructure coupled with hands-on matching supports relationship quality without unnecessary complexity.

The founding and sustained operation of this program would not have been possible without key contributions from Deb McMillen (founding insight); Anitha Chennat, Luellen Fletcher, Kevin Gerrish, Laura Lewis-Tuffin, Heather Olson, Jianjun (J-J) Shen, Sherry Thornton, and Xiaoling Xuei (program design and administration); Phil Hockberger (coach for group mentoring pilot); Nora Dominguez and the University of New Mexico Mentoring Institute (formative guidance); ABRF Executive Director Ken Schoppman (facilitation of the monthly group mentoring forum); our partners at NRMN (Katie Stinson) and sfgMentorNet (Ben Hosie and David Garvie); and the ABRF Executive Board (financial support). Last, but not least, the program was indelibly shaped by the many mentors and mentees who participated, including colleagues from CTLS.

### Lessons Learned

The ABRF Mentoring Program has now been through seven iterations, consistently engaging five to six pairs each time. However, after ten years of operation, some recurring challenges and themes emerge:

1. In the core facility profession, mentee objectives can vary widely. They range from learning new scientific techniques to managing conflict in the workplace; from business skills, marketing, and client relationships to setting up laboratory information management system (LIMS) systems for automated billing and data management; from handling day-to-day operations to pursuing career advancement. **Recruiting and sustaining a large diverse mentor cohort is critical to meeting these needs.** However, it also results in mentors who are not engaged with the program while they wait to be paired, and who may find it hard to re-engage when called upon.
2. In all structures, the program administrators invest substantial energy in establishing new mentor-mentee pairs, hoping to set a good foundation for the relationship. However, this is not sufficient: **the mentor and mentee must also invest in getting to know each other and build trust**. It is easy to underestimate the importance of this step, which may explain the many pairs who did not engage beyond the first couple of meetings.
3. **Mentor training can help** with setting good relationship foundations. The ABRF Mentoring Program has not provided this training consistently or at a useful scale. Many of our participants consider themselves to be experienced mentors not in need of such training. This is an obvious area for improvement in the future.
4. **It is important to regularly assess and reinforce expectations around what participation looks like**. In a program serving core facility professionals, participants are expected to have busy schedules and may need to adjust their involvement, but no one should ghost their partner.
5. Expectations include ensuring that participants respond to surveys designed to confirm the quality of their engagement. **Inconsistent participant feedback impacts mentoring and program quality**. In several cases, mentees and mentors have ignored mid-program surveys and waited until the program’s end to reveal that their partner was not engaged, which is a lost opportunity for everyone involved.
6. **Bigger is not always better**. It was hoped that the virtual mentoring platforms would allow the program to scale while relieving the activity burden on the program administrators. Ultimately, neither platform was viewed as user friendly, with both creating barriers to program engagement; the small, fully manual programs were the most successful. As one program participant stated: “I would suggest pairing people, offering resources, and getting out of the way. The whole point of mentoring is to create a personal connection which may not be enhanced by intrusive software.”
7. **The drawback to fully manual programs is that they rely heavily on the networks and bandwidth of the program administrators.** This can create challenges for responding to initial mentee interest, finding suitable mentors, and adequately following pairs to ensure their quality.

Despite these challenges, the value of the ABRF Mentoring Program to both mentees and mentors is undeniable, as a few survey respondents explained:

1. “I found the experience and advice extremely helpful and was able to give some information and expertise back in my particular field.”
2. “As a Mentor, I found it interesting to see challenges that other people have.”
3. “I thoroughly enjoyed this pairing and will continue building my relationship with my mentor. She has been an invaluable resource for me in my career development.”
4. “This is an excellent resource that many organizations just don’t have. It is especially helpful for those leading core facilities as there just isn’t ‘training’ out there for those positions. This enables people who are in similar situations to guide each other through what can often be unknown territory.”
5. “Mentoring has really taken off in ABRF and has benefited so many participants. As a mentor, I have learned a lot about myself, and it has been gratifying to witness the personal and professional growth of my mentee.”

## CTLS Mentoring Program

The CTLS Mentoring Program^4^ began in 2022, with the aim of supporting professional development for core facility scientific, technical, and administrative staff. One focus of the program was to be as flexible and informal as possible for both mentors and mentees, eliminating barriers to participation. One choice that was made from the onset was to require mentors to be active members of CTLS in good standing. By requiring that mentors be CTLS members, the association maintains a level of oversight and quality control, in particular, by ensuring that mentors abide by the CTLS Code of Conduct.^5^ However, mentees are not required to be CTLS members. This broadens the impact of the program by reducing limitations on participation. As a result, the Mentoring Program is a form of marketing for the association and a mode of entry for potential new members of CTLS. This decision was in keeping with many CTLS professional development activities; for example, the CTLS webinar and workshop program has generally not been limited to CTLS members.

CTLS is fundamentally an association that focuses upon what brings us together as core facility scientists and administrators, rather than separating members by specific technical areas. Transverse topics which benefit all core facility professionals are central to CTLS. While this is evident in the organization of the Mentoring Program, we recognize that due to the nature of core facility career pathways, certain professional development needs can only be satisfied by mentors with specific backgrounds. Thus, in organizing the CTLS Mentoring Program and website, particular attention has been paid to the makeup of the mentor pool, as well as to how mentor expertise is distributed and communicated. While it is not possible to ensure that every technical area, scientific expertise, or administrative role under the very broad core facility umbrella is provided, the program has tried very hard to ensure that the majority of disciplines are appropriately represented. This includes information regarding the administrative duties of the mentors: for example, management (of an individual core), administration (of a whole core facility program), scientific area (e.g., cell biology or immunology), and technical area (e.g., light microscopy or mass spectrometry).

By having these different self-identified characteristics clearly delineated on the program website, prospective mentees could be provided with a menu of alternatives, based upon both their specific areas of expertise and experience as well as their preferred topics of focus for mentorship. Furthermore, we have included a link to each mentor’s LinkedIn profile. This provides prospective mentees with another source of information regarding the mentors that includes more detail than could be provided on the CTLS webpage. Since people normally update their LinkedIn profiles regularly, this means that the Mentoring Program webpage itself does not need to be separately modified as mentor profiles change with new roles, institutions, and skillsets.

The organizational structure chosen for the CTLS Mentoring Program deliberately aims to reduce barriers to participation as well as the imposition of potentially unwieldy requirements for how mentorship might take place. The program does not dictate how frequent meetings should occur, how long they last, or the ultimate duration of the mentorship connection. Some individuals might have one specific set of needs or questions that could be addressed in a single one-hour meeting, while others might want to meet frequently for extended periods. Furthermore, while in practice, the vast majority of mentorship will take place via standard one-hour remote video meetings, this does not preclude other types of communication such as email, or even in-person meetings as appropriate.

Oversight for the program is provided by the Mentorship Focus Group, which is a subsection of the Training Working Group, which is the group within CTLS responsible for coordinating professional development activities. The Mentorship Focus Group serves as the contact point for CTLS members who wish to become mentors, as well as anyone who wants more information regarding the CTLS Mentoring Program. We maintain a log of information related to the program, which includes mentor and mentee names and institutions, dates of first contact, and topics for discussion.

Feedback from CTLS Mentoring Program participants (see the following section) has been extremely positive, including from both mentors and mentees. One interesting observation has been the impact that mentees have had on their mentors. Seeing things through the eyes of those less experienced can provide insights into supervision and strategic management that are not easily obtained through other means. Furthermore, it is very common to be limited by our own specific frames of reference, experiences, backgrounds, and institutions. In many cases, it seems that mentors can learn as much through these interactions as the mentees. That being said, it is extremely important as a mentor to ensure that the focus of the conversation remains on the mentee and the topics that they want addressed.

One main benefit of the CTLS Mentoring Program and others like it is exposure to senior colleagues outside of our institutions. There is a clear link between mentorship and networking. Although institutional colleagues can be an excellent resource for knowledge and advice, it is often helpful to obtain the point of view and opinions of someone outside of our bubble. Broadly speaking, we all face the same challenges, but standard operating procedures (SOPs) and institutional culture can sometimes limit our perspectives, while an alternative approach may emerge more easily with help from an external colleague.

In addition to the Mentoring Program, CTLS has explored a range of mentorship focused events. These have taken place at CTLS congresses as well as in webinars or online workshops. As an example, one online session focused on mentorship, professional development, and work/life balance with talks from Dina Arvanitis (Northwestern University, Chicago) and Louise Gillic (Francis Crick Institute, London). This was an inspirational session with advice on how to juggle work and home demands. The value of such sessions is the opportunity for high-quality speakers to advise an audience of colleagues on the benefits of mentorship. Other CTLS events have included online group-based Peer Mentorship Workshops with approximately 50 attendees, which included topics such as career development, soft skills training, and professional competency training. Feedback from these sessions suggested that this peer approach was less hierarchical and less fixed than traditional mentoring relationships, although of course traditional mentoring can involve colleagues at similar career stages but perhaps in different disciplines. Finally, at the CTLS 2023 Congress in Dublin, four speakers addressed the entire audience in a mentoring-focused session. Their key messages included the value of mentoring to both the mentor and the mentee and how important, even fundamental, these interactions and experiences can become within the context of the careers of highly successful colleagues.

### Feedback from Participants in the CTLS Mentoring Program

Professor Peter O’Toole, University of York, UK:

“I have been a mentor for the CTLS Mentoring Program since its inception, providing support to five different individuals, on a wide variety of topics. It is a key part of being a member of the community. We have all had people who have inspired us, or mentored us, even if they were not aware. I remember starting out with so many questions as facility roles were still novel. By mentoring, we can now help speed up that learning process for others so that they can maximize their impact and fulfill their own potential.

Mentoring also helps my own role. While giving advice, it also provides invaluable time to reflect on my own activities, ways of working, and get feedback from the Mentee with their thoughts. It helps keep me grounded and understand that our careers are ever evolving and that those starting out may be looking for new advice that many of us take for granted but also come with fresh questions that challenge us.

The experience of being a CTLS Mentor has also highlighted activities in my own facility that may have slipped. Practice what you preach! We can talk a good game when offering advice, but that reflection encourages you to check that you and your lab are still enacting what you are talking about. Multiple times, it has helped highlight that activities/best practices can drift over time from how you initially envisioned or designed them, especially as the workload increases over time, but these meetings really help put things back into focus and stop the slippages.”

Doctor Sha Wang, Princeton University, USA:

“In 2021, I joined the Imaging Core Facility at Princeton University following my postdoctoral training. While I was excited about this new role, I also recognized the need for guidance as I transitioned from a traditional research position to becoming a microscopy specialist.

While exploring professional development resources, I discovered the CTLS Mentoring Program and reached out to a potential mentor. I was grateful for his prompt and kind response, and we were able to schedule our first meeting within a week. My mentor’s diverse career path, from earning a PhD in biology, to serving as a faculty member, and later taking on leadership roles in core facilities and research infrastructure, offered a perspective that was both insightful and highly relevant to my own journey.

Over nearly two years of regular Zoom meetings, my mentor was consistently generous with his time and insight. Our conversations ranged from technical topics in microscopy, such as TIRF, STED, and colocalization analysis, to broader discussions about user support, facility management, and long-term career development. He provided thoughtful, practical guidance and was especially helpful in offering a broader perspective, often helping me see challenges from new angles before making decisions. He was also open to a wide range of topics, and when questions extended beyond his immediate expertise, he would offer to connect me with others in the community who could provide additional insight.

This mentorship has been incredibly valuable to me. It has strengthened my technical confidence, broadened my perspective, and helped me develop a clearer vision for my career in core facilities. I am very grateful for the support I received and would warmly recommend the CTLS Mentoring Program to early-career core facility staff seeking guidance and professional growth.”

## CTLS Job Shadowing Programme

While the ABRF and CTLS Mentoring programs focus on structured professional dialogue and networking, the Job Shadowing Programme offers a complementary, practice-oriented model of professional learning based on direct, in-person observation and participation within operational environments.

Modern research infrastructures rarely rely on a single technology or expertise. Instead, core facilities operate at the intersection of multiple methodologies, workflows, and institutional responsibilities. As a result, effective facility management increasingly depends not only on technical competence but also on transversal skills such as communication, coordination, strategic planning, and service design. While cores in certain technical areas at different institutions might possess similar instrumentation, how these cores are employed and the training, support, and management strategies applied within them can vary greatly.

The CTLS Job Shadowing Programme commenced in 2022. Originally named the Shadowing and Staff Exchange Programme, it was developed in recognition of this evolving landscape. It aims to support professional growth by facilitating structured peer-to-peer learning across institutions (see the program description on the CTLS website^6^). The program has rapidly emerged as a key flagship offering to CTLS members, creating immediate and lasting benefits to visitors and hosts.

The program is designed to focus on operational processes, management approaches, and organizational practices rather than on technical training. Visits are not intended to provide hands-on instruction for specific instruments, data acquisition, or analysis pipelines, nor is it intended to support direct participation in research collaborations. Instead, the initiative encourages participants to observe how facilities are structured, how services are organized, and how strategic decisions are implemented in practice. By emphasizing operational learning, the program promotes the exchange of best practices and supports facilities in refining their own management models. Furthermore, by deliberately focusing on core facility management and administration, rather than specialist technical knowledge, the goals and impacts do not depend upon what experiments are being run or which instruments or services are in operation when the visit takes place.

The annual program is launched each year through a call communicated via email and on the CTLS website, and the group provides travel support of up to €1000 (approximately $1140) per visit. Year One (2022) saw 7 visits; 10 took place in 2023 and 12 in 2024. Since 2025, the number of visits has been capped at five annually, and three visits were funded in 2025. This reflects the CTLS Board of Directors’ decision to allocate fewer grants and to prioritize visits centered on operational management rather than technical training. Exchanges typically last between two and five working days, a format designed to balance meaningful exposure to host facilities with minimal disruption to the participants’ operational responsibilities while also allowing the program to support multiple visits each year.

Participation is structured around clearly defined roles. Visitors seek exposure to alternative operational environments, while host facilities provide access to workflows, decision-making structures, and organizational strategies. Oversight of the program is ensured by a dedicated focus group operating within the CTLS Training Working Group. Selection of visits is merit-based and designed to ensure both relevance and impact, while also promoting diversity by limiting funding to one visit per institution within a given year.

The program has thus far supported a range of interaction formats. These include peer-to-peer visits, junior-to-senior visits, and reciprocal exchanges in which the visitor also acted as a host in the same year, thereby exponentially strengthening the benefit of the interaction. This flexibility allows the program to accommodate the diverse needs of the core facility community, spanning different career stages and roles.

In contrast to the Mentoring Program, where mentors must be CTLS members, but mentees may participate without membership, the Job Shadowing Programme adopts the opposite approach. Since 2025, Visitors are required to be CTLS members for at least six months, thus ensuring that participants are actively engaged in the association, whereas hosts are not required to be members. This structure both lowers barriers for host participation and reinforces a reciprocal “give-and-gain” model in which community engagement is progressively strengthened. It also sets up participation as a visitor as a distinct benefit of CTLS membership. Participants may move from visitor to host, from host to focus group member, and eventually into broader participation within CTLS activities including leadership roles within the association.

The impact of the program extends beyond individual visits. Participants frequently become ambassadors for CTLS within their institutions, strengthening the visibility of the association while simultaneously expanding professional networks across the research infrastructure landscape. By enabling direct observation of alternative operational models, the program fosters collaborations, supports knowledge transfer, and encourages strategic reflection on facility practices.

As with the CTLS Mentoring Program, the CTLS Congress has played an important role in sustaining the Job Shadowing Programme. This has provided opportunities to recruit hosts, visitors, and focus group members. Informal discussions during congress meetings frequently evolve into formal shadowing visits, demonstrating the importance of community gatherings in supporting professional development initiatives.

As part of the program’s evolution in 2026, targeted measures are being implemented to strengthen its visibility and accessibility. These include improving the visibility of committed hosts to reduce barriers for potential applicants who may be less confident about initiating contact, as well as expanding the diversity of the focus group to better reflect the breadth of expertise, institutions, and disciplines across the core facility landscape.

Looking ahead, the Job Shadowing Programme is envisioned not only as a mobility scheme but also as a strategic tool for shaping the next generation of core facility professionals. By embedding peer learning, operational transparency, and cross-institutional exchange into everyday practice, shadowing can help cultivate a more connected, reflective, and resilient research infrastructure community.

### Visit Testimonial 1

**Figure 1.**
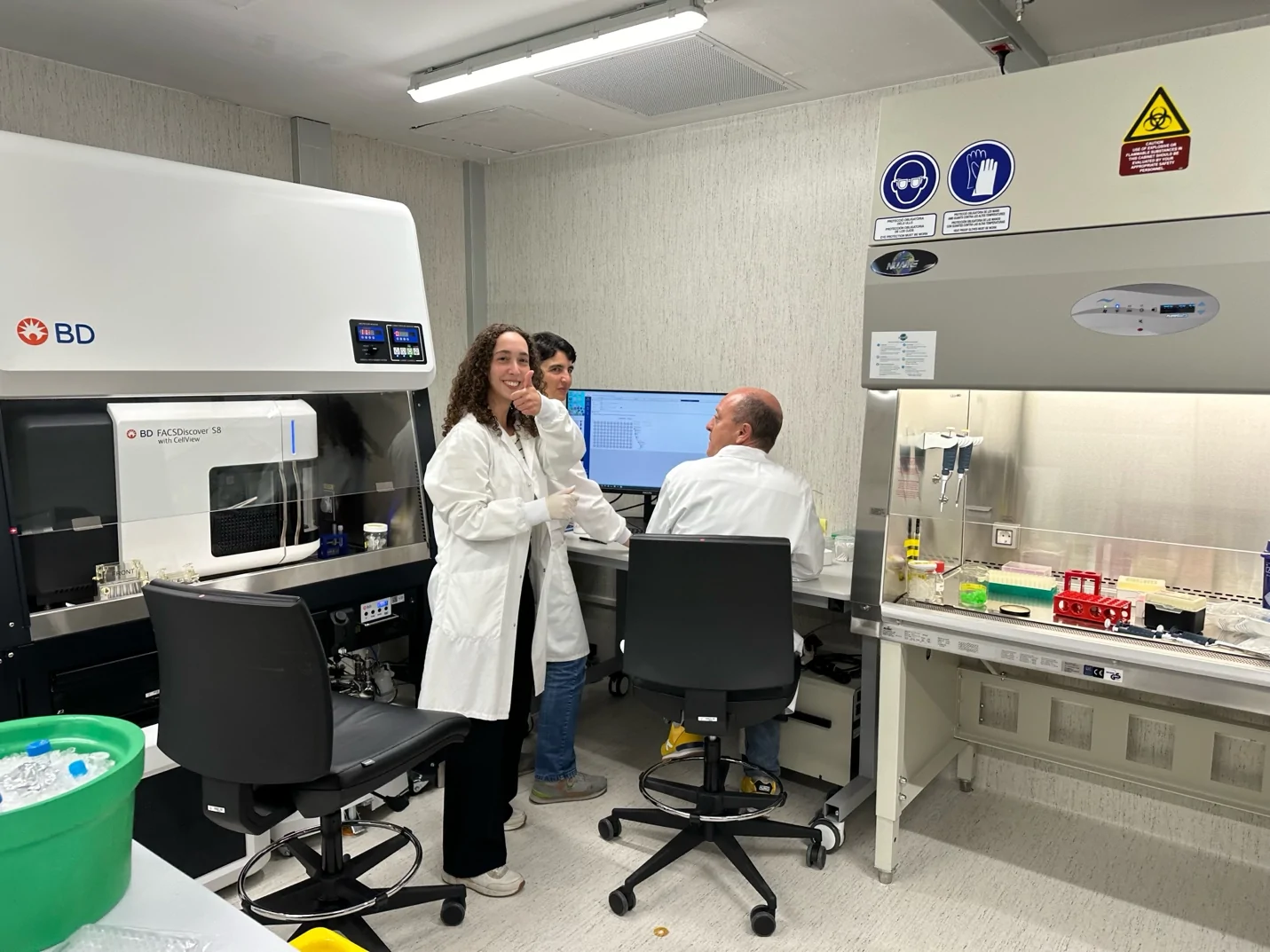
**CTLS Job Shadowing Programme, Visit Testimonial 1**. Beatriz Teixeira (left) and Dr. Oscar Fornas (seated) during Beatriz’s visit to the Centre for Genomic Regulation (CRG) and Pompeu Fabra University (UPF) Flow Cytometry Core Facility in Barcelona, Spain.

Beatriz Teixeira, Senior Laboratory Research Scientist at the Francis Crick Institute in London (UK), visited Oscar Fornas, Head of the Flow Cytometry Core Facility at the Centre for Genomic Regulation (CRG) and Pompeu Fabra University (UPF) in Barcelona (Spain) (see Figure 1).

Beatriz Teixeira:

“The CTLS Job Shadowing Programme allowed me to get a new perspective on how a high-quality research institute, like the CRG, and particularly Oscar Fornas’ team from the Flow Cytometry Core Facility, manage a wide spread of scientific applications across their core unit. It inspired me to work on collaborations that I believe could greatly benefit the Flow Cytometry Unit at the Francis Crick Institute and will help me grow as a Flow Core Staff. Getting to know the team was a great opportunity to exchange experiences and know-how on flow cytometry as well as management strategies and platforms for team organization. It was very enriching to see how the diverse technologies available at the CRG contribute to the scientific development of research, which prompted me to think of ways these could translate into our institute. During my visit, I had many important discussions about how and where the field was moving, ways to contribute to the scientific community, and the role core facilities have on research institutes. I really enjoyed my time in the CRG and feel that this whole experience left me with a sense of agency about my future and what I want to become professionally.”

Oscar Fornas:

“The experience of participating as a host in the Shadowing and Staff Exchange Programme has been very rewarding. The learning has been bidirectional. We have learned from Beatriz, and she has been able to see how another unit like theirs is managed. Different points of view, objectives, and ways of working are not always the same. This is exactly why you learn a lot from how others manage teams and infrastructures similar to your own.

Without a doubt, it has been a great experience and highly recommended. I would be happy to repeat the experience and even send a member of our team to visit another core unit.”

### Visit Testimonial 2

**Figure 2.**
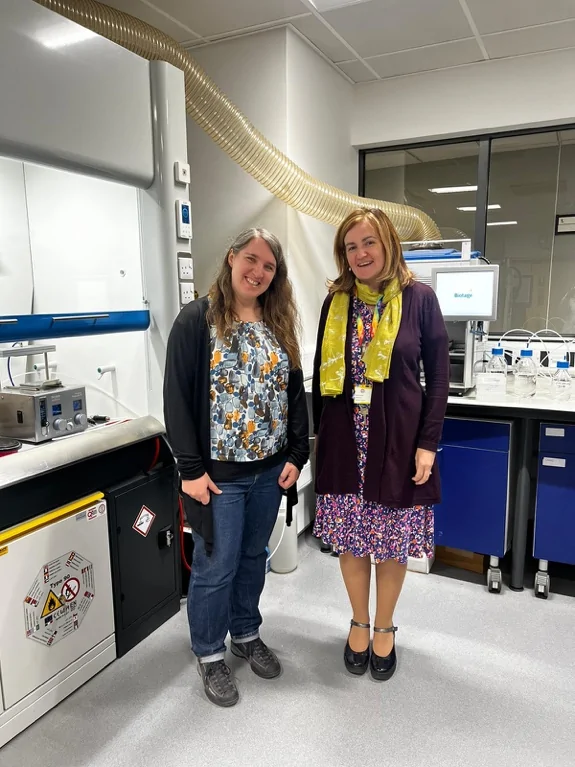
**CTLS Job Shadowing Programme, Visit Testimonial 2.** Dr. Maria Doppler (left) and Dr. Natalie Homer (right) during Maria’s visit to the Mass Spec Core Facility at the University of Edinburgh.

Maria Doppler, Senior Scientist at the Universität für Bodenkultur Wien (BOKU) University Vienna (Austria), visited Natalie Homer, Deputy Director of the Mass Spectrometry Core Research Facility, School of Neurological and Cardiovascular Sciences at the University of Edinburgh (UK) (see Figure 2).

Maria Doppler:

“During my one-week shadowing stay in Edinburgh, I had the opportunity to visit Natalie Homer in her Edinburgh Clinical Research Mass Spec Facility lab and gain valuable insights into the workings of her core facility. I met the team, observed their daily lab activities, and saw how they interact with users and manage workflows. This experience provided me with a practical understanding of their operations, including discussions on results and showcasing the lab to students. Additionally, I attended an introductory workshop on a software tool that came with a new instrument. Here, my experience in the field of untargeted metabolomics was beneficial and I could share some tips and tricks on how we carry out our experiments in the BOKU Core Facility Bioactive Molecules: Screening and Analysis. A significant part of my stay involved exchanging with Natalie on key managerial topics such as cost calculation, team leadership, task management, reporting processes, and strategies for acquiring new instrumentation. These discussions were highly beneficial, giving me practical tools for improving the management of my own facility. Another major aspect was visiting other core facilities in Edinburgh, where I toured a total of 10 different facilities. I was warmly welcomed by their teams and had the opportunity to exchange ideas on a wide range of topics related to running a core facility. The open-mindedness and expertise of the people I met provided me with a wealth of new insights. Overall, the visit gave me valuable input, especially from their long-standing experience, which I can apply to further advance the BOKU Core Facility Bioactive Molecules: Screening and Analysis, and I’m already looking forward to putting the new input into practice.”

Natalie Homer:

“Taking part in the Shadowing and Staff Exchange Programme is beneficial to both the member of staff who travels and to those they are visiting. For those being visited, it allows you to reflect on what it is you do to make your core facility work. To think about what the challenges are, to demonstrate how you have set up your facility and how you interact with researchers and students. It also gives the opportunity to learn from the member of staff visiting. Of great benefit is that my whole team had the chance to meet Maria, which meant that they had time to ask questions and learn from the experience too.”

## Conclusion

As the core facility career pathway continues to expand and evolve, associations such as ABRF and CTLS must continue to provide professional development activities for their members. This paper has highlighted two examples that have resulted in durable member benefits, namely mentorship and job shadowing programs (see the programs summarized in Table 1).

**Table 1.** Comparative program summary

**Program**	**ABRF Mentoring**	**CTLS Mentoring**	**CTLS Job Shadowing**
**URL**	https://form.jotform.com/242735284263156	https://ctls-org.eu/community/ctls-mentors	https://ctls-org.eu/community/job-shadowing-programme
**Point of Initiation**	Mentee fills out info form and then is connected to mentor by program facilitator	Mentees reach out directly to potential mentors	Applications from visitors and hosts submitted during open call period
**Organization**	Structured relationship between mentor and mentee; mentee should have specific goal or topic in mind	Flexible program focused on professional development, scientific fields, and core facility management	Maximum award of €1000 (approximately $1140) to the visitor to cover travel expenses for visit to learn about core facility management and operations
**Program Length**	3-6 months	Open-ended	1 week maximum
**Oversight and Assessment**	Program facilitator and ABRF Code of Conduct	CTLS Mentorship Focus Group and CTLS Code of Conduct	Job Shadowing Focus Group and CTLS Code of Conduct
**Responsible Body**	ABRF Career Development Committee	CTLS Training Working Group	CTLS Training Working Group
**Financial Support**	No expenses incurred with current program; funding for prior program iterations provided by the ABRF	No expenses incurred	Funding provided by CTLS Association
**Restrictions**	Mentee must be ABRF member	Mentor must be CTLS member	Visitors must be CTLS members. Maximum one award to the same institution per year, with five awards total per year

In both cases, these critically important activities have evolved from their initial structure to better serve association membership. The development of these programs has been deliberate, demonstrating clear organizational commitment to providing useful assistance to core facility staff. The program structures that have emerged reflect the differences in the scale, scope, and organizational structure of the two groups while also highlighting similarities in the needs of their respective memberships.

One important challenge with these types of professional development programs is ensuring that their structure supports the needs of their users. To this end, all three programs solicit varied types of participant feedback. The ABRF Mentoring Program has included participant surveys in all program iterations. However, the utility of survey responses is only as good as the quantity and quality of the feedback received. This is something the ABRF continues to work to improve. The CTLS Mentoring Program maintains a voluntary group log sheet for mentors to share information regarding their experiences. However, this primarily serves as a repository for data such as mentee name, date of first contact, and topics discussed. CTLS is working on ways to make the program more proactive with oversight and strategic planning and to increase communication between the association and the mentors/mentees.

By comparison, the CTLS Job Shadowing Chair collects feedback from both visitors and hosts shortly after each visit through a mandatory final report, completion of which is required to receive the award. This approach facilitates an efficient, systematic collection of feedback and helps the association continuously assess and reinforce the value of the initiative while identifying opportunities for improvement. In the future, expanding follow-up surveys could further help assess the long-term impact of the initiative.

Fundamental to the programs described here is the idea of connection: formally creating, fostering, and nurturing relationships; recognizing that people (core scientists, technical staff, administrators) are the greatest resources available for learning, soft skills development, and career growth. Once a solid relationship has been established, program infrastructure can step out of the way and let that relationship bloom. Yet, the programs maintain feedback channels with their participants to enable the evaluations needed for improvement of the program itself. Moving forward, it will be interesting to watch how the activities described here continue to grow and change and maybe spin off other types of professional development activities to benefit ABRF and CTLS members.

### Financial Support/Conflict of Interest

The authors declare that they have no conflict of interest.

### Study Approval

This manuscript does not involve research on human or animal subjects.
